# Microfluidic Devices for Precise Measurements of Cell Directionality Reveal a Role for Glutamine during Cell Migration

**DOI:** 10.21203/rs.3.rs-2799430/v1

**Published:** 2023-05-03

**Authors:** Nil Gural, Daniel Irimia

**Affiliations:** Massachusetts General Hospital; Massachusetts General Hospital

## Abstract

Cancer cells that migrate from tumors into surrounding tissues are responsible for cancer dissemination through the body. Microfluidic devices have been instrumental in discovering unexpected features of cancer cell migration, including the migration in self-generated gradients and the contributions of cell-cell contact during collective migration. Here, we design microfluidic channels with five successive bifurcations to characterize the directionality of cancer cell migration with high precision. We find that the directional decisions of cancer cells moving through bifurcating channels in response to self-generated epidermal growth factor (EGF) gradients require the presence of glutamine in the culture media. A biophysical model helps quantify the contribution of glucose and glutamine to cancer cell orientation during migration in self-generated gradients. Our study uncovers an unexpected interplay between cancer cell metabolism and cancer cell migration studies and may eventually lead to new ways to delay cancer cell invasion.

## Introduction

Cancer cell migration and cancer cell metabolism are two hallmarks of cancer. While cancer cell migration is responsible for forming metastases that cause death in 90% of cancer patients [1], cancer cell metabolism is vital for cancer cell proliferation and tumor growth [2]. However, despite their importance, the metabolism and migration of cancer cells are most often studied independently. It is commonly assumed that cells can produce the energy needed for migration by using various substrates [3, 4]. and, as such, cell migration is unlikely to be affected by the energy source in the cell.

Among the various metabolic substrates employed by cancer cells, glutamine is the focus of intense research and is regarded as a potential target in anti-cancer treatments [5]. However, the role of glutamine in cell migration remains controversial. A few recent studies suggest that glutamine is needed for cell migration [6, 7], while one other study suggests that the lack of glutamine enhances cancer cell migration [8]. One of the major challenges in probing the relationship between glutamine metabolism and cell migration is the lack of specificity of the traditional cell migration assays [9]. Specifically, the assays employed for testing cell migration are sensitive to changes in cell proliferation rates [10], which are also altered by changing glutamine concentrations. Thus, the studies of cell migration at different glutamine concentrations are confounded by the combined changes in cell migration and proliferation. Isolating the relationship between glutamine metabolism and cell migration from potential confounding factors requires a new assay for cell migration.

Our understanding of cancer cell migration has benefited significantly in the past decade from the emergence of microfluidic technologies. Microfluidic tools helped elucidate the contribution of self-generated gradients of epidermal growth factor (EGF) in cancer cell orientation during cancer migration [11] and probe the molecular signaling pathways during the epithelial-to-mesenchymal transition [12]. The applications of microfluidics tools are continuously expanding and today include many tools for the precision measurements of cell migration in conditions that replicate in vivo mechanical confinement [13], the isolation of rare cancer cells from circulation [14], and the in vitro replication of immune-cancer cell interactions [15].

Here we design microfluidic devices with bifurcating channels to study the directionality of cancer cells during the migration in self-generated EGF gradients with high precision. The device helped reveal that glutamine contributes to the orientation of cancer cells through these channels. While the migration of cancer cells through channels is related to *in vivo* metastatic potential, our results could have implications for uncovering new strategies aimed at reducing cancer invasion by interfering with the migration of cancer cells.

## Materials and Methods

### Microfluidic Devices:

Microfluidic devices were fabricated using standard microfabrication techniques. Briefly, a 2 mm thick layer of poly dimethyl siloxane (PDMS, Dow Corning, Midland, MA) was cast on a silicon wafer with photolithographic features. The silicon wafer supports three photolithographic layers. The first, 7 μm thick layer, defines the bifurcating channels through which the cells migrate. A second, 12 μm-thick layer, defines a side channel inside which cells line up before moving into the bifurcating channels. The side channel is designed such that it maintains low cell density per unit surface and lines up the cells at the entrance of the channels at the beginning of each experiment. A third, 50 μm thick layer, defines the loading channels and the connections to the outer compartments of the device. A central well is cut with a 0.75 mm hole punch, connecting the loading channels and the inlet port. Finally, each device is cut using a 5 mm hole punch. Forty-eight devices are exposed to oxygen plasma for 20 seconds (March, Concord, MA) and manually placed, in pairs, on a 24-well glass-bottom plate (Mattek) and baked for 6 minutes at 70°C.

Immediately after bonding, 2 μL of 30 μg mL-1 collagen IV solution (Sigma Aldrich, St. Louis, MO) in Phosphate Buffer (PBS, Life Technologies, Grand Island, NY) is pipetted onto the center well, priming the devices. After a few minutes, when the channels are coated with collagen, each well is filled with approximately 1 mL of media with corresponding epithelial growth factor (EGF, Lonza Walkersville, Hopkinton, MA), glutamine (Sigma Aldrich, St. Louis, MO), and Dimethyl 2-oxoglutarate (AKG, Sigma Aldrich, St. Louis, MO) concentrations. The entire plate is then placed under a vacuum for 5 minutes to facilitate the removal of bubbles from the central well.

### Cell Preparation:

The Non-Small Lung Carcinoma Cell Line (PC9ZD, a gift from Dr. Sri Sharma at the MGH Cancer Center) is cultured in RPMI media (Lonza Walkersville, Hopkinton, MA), supplemented with 10% Fetal Bovine Serum (FBS, Life Technologies, Grand Island, NY), 1% Penicillin/Streptomycin (Life Technologies, Grand Island, NY), 2mM Glutamine and 1mM Sodium Pyruvate (Sigma Aldrich, St. Louis, MO) in a humidified atmosphere of 5% CO2 in the air.

Human mammary epithelial cells (HMEC, Lonza Walkersville, Hopkinton, MA) are cultured in Mammary Epithelial Growth Media (MEGM, Lonza Walkersville, Hopkinton, MA) supplemented with bovine pituitary extract, EGF, hydrocortisone, insulin and gentamicin/amphotericin-B (Lonza Walkersville, Hopkinton, MA), following standard protocols. For the directionality experiments, the microfluidic devices are filled with DMEM glucose and glutamine-free media supplemented with glutamine, glucose, or both.

Prior to each experiment, the tumor cells grown in culture flasks are rinsed with 1X PBS, and detached from the flask surface by incubation with 1.5 mL Accutase (Sigma Aldrich, St. Louis, MO) for 4 minutes. The cell release solution is then neutralized by the addition of 3.5 mL growth media. Epithelial cells are washed with 5 ml of HEPES Buffered Saline Solution (HEPES-BSS, Lonza Walkersville, Hopkinton, MA) and detached from the surface by incubation for 2–6 minutes after the addition of 2 mL Trypsin/EDTA (HEPES-BSS, Lonza Walkersville, Hopkinton, MA). After incubation, the cell release solution is neutralized by the addition of 4 mL of Trypsin Neutralizing Solution (TNS, Lonza Walkersville, Hopkinton, MA).

The cell suspension is then separated into tubes filled with growth media. The tubes are centrifuged at 1000 rpm for 5 min at 25°C. After centrifugation, the cells are re-suspended at a concentration of ~1 million per milliliter in assay media formulations for each experiment. After vacuum, media in each well of the plate is removed, allowing only the central side channels and bifurcating channels to be coated with the media. Suspensions of cells are loaded into each collagen-coated device by placing a 2–3 μL droplet of cells onto the central well. The plate is observed under a microscope until the side channels are filled with cells. Finally, 1 to 1.5 mL of assay media is added to each well to fully submerge the devices in the media.

### Assay Media:

Assay media is prepared by adding 1ng/mL EGF and 1 mM sodium pyruvate into DMEM supplemented with 10% dialyzed FBS (Life Technologies, Grand Island, NY) and 1% penicillin/streptomycin. Different DMEM formulations with varying concentrations of glutamine and glucose are employed for the experiments (catalog numbers 11965 (glutamine, glucose), 11054 (no glutamine, low glucose), 11966 (glutamine, no glucose), and A1443001 (no glutamine, no glucose) - Thermo Fisher). For some studies, 2 mM AKG is added to the media.

### ATP measurements:

Luminescence ATP Detection Assay System (ATPlite, Perkin Elmer, Waltham, MA) is used to monitor the ATP usage of cells in different media conditions. Cells are plated in a white, opaque 96-well plate (Corning, Tewksbury, MA) and left in 100 μL of their corresponding media for 1 hour. Then the assay is performed according to the recommendations of the manufacturer.

### Image Analysis:

Time-lapse imaging is performed with a fully automated Nikon Ti-E microscope fitted with the perfect focus system and an environmental chamber maintained at 37°C. Images are captured at 8 locations for each device every 20 minutes for 30 hours. Cells move through the channels from a central compartment with cells towards the outer compartment without cells. At each bifurcation, cells could turn towards the dead-end or the through path. In addition, cells can turn back towards the cell compartment. To quantify the directional decisions, we counted the cells that chose the through path at each of the five bifurcations. We normalize their number to the total number of cells to enter the channels.

### Statistical Analysis:

Each directional choice made by moving cells through the channels in the device is categorized as a binary choice, either through or dead-end. Up to five choices are quantified for the cells entering the channels with five bifurcations. Cells that made dead-end choices are not followed further even if they turn back and choose a through path. Only the first cells to enter each channel and reach at least one bifurcation are evaluated. Whenever a cell inside the channel undergoes division, it is excluded from the count.

We calculate an orientation factor (*b*) by first counting the decisions made by cells passing through each of the five bifurcations. We then normalize these counts to the total number of cells to enter the channels. We also develop a model that calculates the fraction of cells passing through each of the bifurcations when the orientation at all five bifurcations was uniform along the channel and orientation factors between 0.5 (random decisions) and 1 (always through the channel). We calculate the experimental *b* value for which the squared differences between the model and experimental measurements reach a minimum. We also test if the orientation factor changes along the channel are significant between the first and subsequent bifurcations using a chi-square goodness of fit test. Orientation factors from different experimental conditions are compared using ANOVA statistics. Differences are considered statistically significant for p ≤ 0.05.

## Results

**Figure 1:**
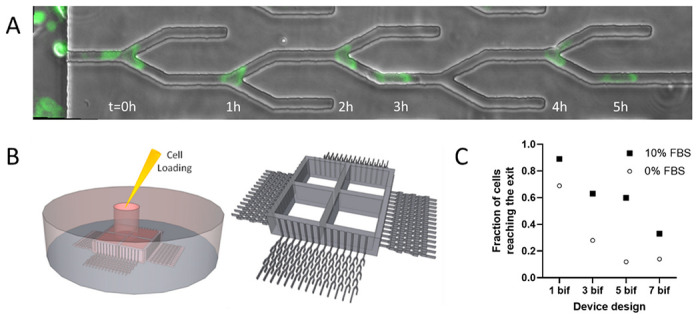
Microfluidic assay for precision measurements of directionality during migration. A. The location of a fluorescently tagged PC9 cell at different times along one channel with five bifurcations is captured in overlapping images. In this example, the cell avoids the dead ends and exits the channel at 5 hours. Scale bar is 20 μm. B. Cells are loaded through a middle compartment. Cells sediment along the side channels, within 200 μm of the entrance to the bifurcating channels. C. We calculated the fraction of cells that pass through channels with 1 to 7 successive bifurcations in the presence and absence of serum in the media (filled squares vs. open circles, respectively); N = 266, 33, 67, 27 cells in devices with 1, 3, 5, 7 bifurcations in the presence of serum; N = 228, 43, 27, 14 cells in devices with 1, 3, 5, 7 bifurcations in the absence of serum).

We designed a microfluidic tool that enables high-precision analysis of the directional choices made by moving cancer cells (Fig. 1). Inside these devices, cells are loaded through a small inlet and driven through high-aspect-ratio channels sideways to four side channels. These side channels are connected to the migration channels and, through these, to an external reservoir of fresh media. After loading, the cells travel through the high aspect ratio channels and settle at the entrance of the migration channels. While the cross-section of the migration channels is smaller than that of the suspended cells, the settled cells are trapped at the entrance. The cell trapping also restricts the fluid flow during cell loading from the inlet to the migration channels without cells, ultimately helping distribute the cells uniformly through the device at the entrance of the migration channels. After adhering to the bottom of the device, the epithelial cells start moving toward the external chamber. The moving cells are confined in channels and pass through successive where they could enter either dead-end or through channels (Fig. 1A). The design of the device includes a narrow cell loading chamber such that cells introduced into the device are within 200 μm from the entrance to the migration channels, increasing the overall yield (Fig. 1B).

We compared the yield of cells passing through channels with one, three, five, and seven bifurcations. We reasoned that increasing the number of bifurcations could increase the precision of evaluating each cell’s performance by increasing the number of repeated observations in similar conditions along the channel. However, while the number of cells reaching the end of channels after a large number of bifurcations decreases, the contribution of later bifurcations to the information generated by the devices also decreases. Moreover, the added migration distance also extends the duration of experiments. Thus, for the purpose of design optimization, we compared the throughput of four designs with 1, 3, 5, and 7 bifurcations. We observed a significant drop in the cellular yield between devices from 5 and 7 bifurcations per channel (Fig. 1B). Thus, we chose for this study to use devices with five bifurcations, which cells traverse in approximately 6 hours.

**Figure 2:**
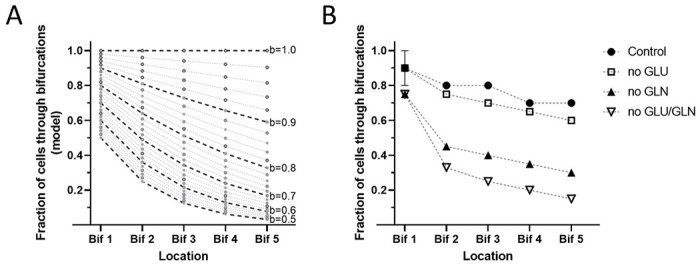
Fraction of cells moving through channels with bifurcations. A. Calculated fractions of cells passing through each bifurcation of a five-bifurcations channel, assuming constant ‘orientation factor’ throughout the channel. Calculated values for orientation factors between 0.5 (random decisions) and 1 (always through the channel) are represented in 0.02 increments. B. Experimental results show the fraction of cells passing through each bifurcation. PC9 cells in control media (filled circles), media with no glucose (empty circles), media with no glutamine (filled triangles), and media with no glutamine or glucose (empty triangles, N = 416, 531, 261, 166 cells observed, respectively; N=6 experiments). The dotted lines are a guide for the eye.

We quantified the bias of the cells moving through successive bifurcations as orientation factor b and calculated the fraction of cells through each bifurcation, assuming independent decisions and uniform probability of cells taking the through path (Fig. 2A). We represented the changes in the fraction of through cells for orientation factors b with values between 1 and 0.5. A value of the orientation factor b = 1 would indicate cells that are biased towards the through channel all the time, and b = 0.5 cells that make random decisions.

For each set of experimentally determined values, we determined the value of the orientation factor b that best fits the predicted and experimental values. We then employed the orientation factor to compare the performance of cells in different conditions. We found that PC9 cells in growth media display a strong bias towards the through channels. Out of 796 cells moving through devices, 459 reached the end, representing 58% of the cells (Fig. 2B). Our calculated orientation factor for these cells was b = 0.9. If cells made random choices at each bifurcation (b = 0.5), we would have expected 3.1 % of cells to go through the entire channel. The twenty-fold difference in the fraction of cells exiting the channel confirmed that a significant bias towards the through channels exists for PC9 cells in media.

We compared the cells moving through the channels with bifurcations in the presence and absence of glucose and glutamine. We found that out of 580 cells to enter the channels in the presence of media with dialyzed FBS, DMEM, glucose (5.5 mM), and glutamine (2.0 mM), 358 will go through all five bifurcations (b = 0.92). If glutamine is absent from the media, the orientation decreases significantly, and only 140 of 482 cells go through (b = 0.70, Fig. 2B). In the absence of glucose, the fraction of cells to go through is comparable to that in the controlled media (b = 0.90). The effect of removing both glucose and glutamine from media is comparable to the removal of glutamine alone (b = 0.70).

**Figure 3:**
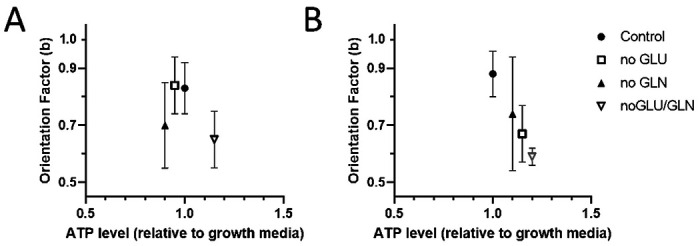
Orientation factor and cellular ATP levels. A. ATP levels measured in PC9 cells are comparable for all media formulations (N = 767, 312, 494, 316 cell trajectories analyzed for control, no glutamine, no glucose, and no glutamine no glucose conditions, respectively; N=4-6 experiments). B. ATP levels measured in HMEC cells did not change significantly in media with no glucose or glutamine, whereas the orientation factor is decreased (N = 249, 96, 75, 243 for control, no glutamine, no glucose, and no glutamine no glucose conditions, respectively; N=2-3 experiments). Bars represent standard deviations.

We measured the ATP levels and cell speed for PC9 and HMEC cells in the presence of glucose and glutamine in media. We found no significant differences in the ATP levels in the absence of glutamine and glucose and no correlation between the ATP levels and the changes in orientation (Fig. 3A). We also measured the levels of EGF in the media of cells cultivated in the presence and absence of glutamine. We found no significant differences in EGF levels (data now shown).

We also analyzed the effect of glucose and glutamine on migrating non-tumor human mammary epithelial cell line (HMEC). Using a DMEM media as a reference, we calculated that the orientation factor (b = 0.88) decreased both in the absence of glucose (b = 0.67) and the absence of glutamine (b = 0.74). When both glucose and glutamine are absent, an even smaller number of cells migrate, and none of them reaches the end of the channel (b = 0.59). We found that the ATP levels in HMEC cells did not change significantly when glucose and glutamine were removed and increased the most when both glucose and glutamine were removed (Fig. 3B). The interchangeable role of glutamine and glucose in non-tumor epithelial cells (HMEC) and the unique role of glutamine in tumor cells (PC9) point to some contributions from the Krebs cycle. While PC9 cells cannot use glucose in the Krebs cycle, non-tumor cells could use both nutrient sources.

**Figure 4:**
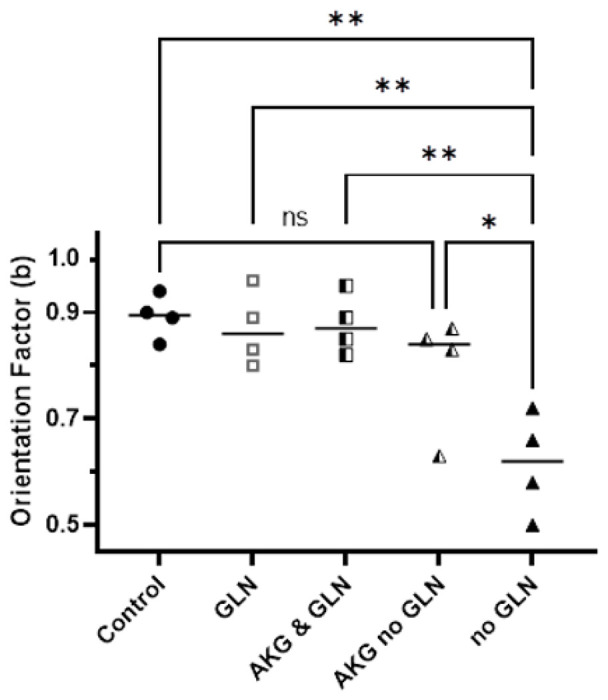
Alpha-ketoglutarate restores cell orientation at bifurcations. The orientation of PC9 cells navigating through bifurcating channels, which is decreased in the absence of glutamine, can be restored by the addition of alpha-ketoglutarate. Control media with glucose and glutamine (full circles, N = 327 cells), glutamine and no glucose (empty squares, N = 276 cells), alpha-ketoglutarate and glutamine (half-filled squares, N=337 cells), alpha-ketoglutarate and no glutamine (half-filled triangles, N=279 cells), and no glutamine (full triangles, N=120 cells). N=4 experiments, each experiment including all conditions. The significance of differences was evaluated using ANOVA, * p <0.05, ** p<0.001.

We tested the effect of alpha-ketoglutarate (AKG) on the orientation factor of PC9 cells. We reasoned that AKG could enter the Krebs cycle as an alternative to glutamine in cells that could not use glucose. We found that the orientation factor is corrected by AKG (b = 0.79, Fig. 4). The cells exposed to a combination of AKG and glutamine displayed a modest improvement in orientation (b = 0.88), suggestive for an interchangeable role of glutamine and AKG in cell directionality.

## Discussion

We designed microfluidic devices with bifurcating channels to test the directionality of cells during migration. The devices with five bifurcations were determined to be optimal for measuring the contribution of glutamine to directional decisions in cancer epithelial cells. The technical advance described here includes the design of multiple bifurcations in series along the devices. This design element is critical for enhancing the precision of the measurements by exposing the moving cells repeatedly to the same directional challenge. Similar to other devices for probing cancer cell migration, the devices employed in this study rely on gradients of epidermal growth factor (EGF) generated between the cancer cells in the loading channel and the fresh media in the external chamber, as described before [11, 16–18]. Innovative design elements also include the use of a small inlet and high aspect ratio channels for positioning the cells to the entrance of the channels, increasing the yield of the devices by minimizing the distance the cells have to travel before entering the migration channels. The simple computational model developed to average the information from multiple experiments helped summarize the effect of media into one index and enhanced the precision of the measurements.

Glutamine is the most abundant amino acid in the body (at around 0.6 mM in circulation). Glutamine is essential in the growth and division of cancer cells [19], energy production [20, 21], lipid synthesis under hypoxic conditions [22], ribonucleotide synthesis [23], and protects the cells under oxidative stress [24]. Unlike the prokaryotic cells, which have receptors for glutamine and other amino acids and employ them for directional migration towards energetic substrates [25], no glutamine receptors have been found in mammalian cells. Glutamine enters the cells and is also transported across the mitochondria membranes facilitated by glutamine transporter molecules.

When evaluating the role of glutamine in the directionality of cell migration, a major confounding factor can be cell proliferation, for which glutamine dependence is well known. This situation is due to the fundamental limitations of current cell migration assays. The ubiquitous transwell migration assay evaluates migration indirectly and has poor precision. A major confounding factor is the proliferation of the cells on both sides of the membranes, which could double their numbers in the ~ 24 hours duration of the assay. While cell proliferation is already known to depend on the presence of glutamine [26–28], it becomes difficult to decouple the two and make rigorous determinations on cell migration using the transwell assay. Similarly, the results of the wound healing assays also have poor precision for measuring cell migration. The cells in the monolayer can not only move but also increase their spreading area at the edge of the wound and proliferate in the monolayer. All these activities could reduce the size of the wound over the 24–72 hours duration of the experiments. One could not distinguish between wound closing due to the proliferation in the remaining layer, increasing cell area, or cell migration [9, 10]. When these processes that take place at the same time also share the dependence of a common factor, i.e., glutamine, the interpretation of the results becomes very difficult.

A potential mechanistic insight into the role of glutamine in the directional migration of cancer cells may emerge from our observations that the absence of glutamine is bypassed by adding AKG to media. This suggests a relationship between a complete Krebs cycle and the directional migration of cancer cells. Further supporting this relationship, our study shows that in non-tumor cells which could use both glucose and glutamine in the Krebs cycle, removing either glucose or glutamine does not alter orientation, and only removing both does. A role of the Krebs cycle in cell orientation appears consistent with studies showing that the positioning of the mitochondria at the leading edge of moving cancer cells correlates with their directional migration abilities [29, 30] as well as with studies supporting a link between metabolism, mitochondria, and motility in cancer cells [31, 32]. While our measurements are focused on the trajectories of the first cells to enter each channel, we exclude the potential role of other signaling elements released by moving cells e.g. extracellular vesicles, membrane debris, or secreted molecules, which could bias the migration of follower cells.

At this time, we can only speculate that the role of glutamine in cancer cell orientation is relevant to cancer metastases. However, situations of low glutamine concentration may exist *in vivo*. For cells at the center of a tumor without proper vascularization, the local decrease in glutamine concentration is caused by the imbalance between high glutamine consumption rates and limited supply [33]. Further contributing to lower local glutamine levels trapping may be the white blood cells infiltrating the tumors, which are also known to consume large amounts of glutamine for their metabolism [34–36]. The emerging relationship between glutamine and directional migration suggests an interesting scenario of low glutamine concertation that may help trap the cells inside the tumors, delaying the spreading of cancer cells away from a tumor. Further studies will be needed to determine if these mechanisms occur *in vivo* and eventually measure their contribution to cancer metastases in patients.

## Data Availability

The datasets used and/or analysed during the current study available from the corresponding author on reasonable request.
